# Porcine Models of Pancreatic Cancer

**DOI:** 10.3389/fonc.2019.00144

**Published:** 2019-03-12

**Authors:** Katie L. Bailey, Mark A. Carlson

**Affiliations:** ^1^Department of Surgery, University of Nebraska Medical Center, Omaha, NE, United States; ^2^Department of Surgery and Department of Genetics, Cell Biology and Anatomy, University of Nebraska Medical Center, Omaha, NE, United States; ^3^Department of Surgery, VA Nebraska-Western Iowa Health Care System, Omaha, NE, United States

**Keywords:** pancreatic cancer, swine, porcine, transgenic, KRAS, p53

## Abstract

Pancreatic cancer is the fourth most common cause of cancer-related deaths in both men and women. The 5-year survival rate for metastatic pancreatic cancer is only 8%. There remains a need for improved early diagnosis and therapy for pancreatic cancer. Murine models are the current standard for preclinical study of pancreatic cancer. However, mice may not accurately reflect human biology because of a variety of differences between the two species. Remarkably, only 5–8% of anti-cancer drugs that have emerged from preclinical studies and entered clinical studies have ultimately been approved for clinical use. The cause of this poor approval rate is multi-factorial, but may in part be due to use of murine models that have limited accuracy with respect to human disease. Murine models also have limited utility in the development of diagnostic or interventional technology that require a human-sized model. So, at present, there remains a need for improved animal models of pancreatic cancer. The rationale for a porcine model of pancreatic cancer is (i) to enable development of diagnostic/therapeutic devices for which murine models have limited utility; and (ii) to have a highly predictive preclinical model in which anti-cancer therapies can be tested and optimized prior to a clinical trial. Recently, pancreatic tumors were induced in transgenic Oncopigs and porcine pancreatic ductal cells were transformed that contain oncogenic KRAS and p53-null mutations. Both techniques to induce pancreatic tumors in pigs are undergoing further refinement and expansion. The Oncopig currently is commercially available, and it is conceivable that other porcine models of pancreatic cancer may be available for general use in the near future.

## Background: Pancreatic Cancer

Pancreatic cancer (PC) is the twelfth most common cancer worldwide, with 460,000 new cases reported in 2018 (1). In the United States alone, it is estimated there will be 55,000 new cases of PC diagnosed in 2018, and 44,000 people with succumb to the disease (1). Over the last 40 years the demographic most affected by PC has been white men over the age of 60 (2). One of the main risk factors associated with development of PC is smoking, which is associated with a two-fold increase in incidence (2). Even with advances in our understanding of PC, the incidence has been rising ~0.5% each year over the last 10 years (2), and the 5-year survival rates in localized, regional (nodal spread), or metastatic disease have been 29, 11, and 2.6%, respectively (1–3). By 2030, PC is expected to be the second-leading cause of cancer mortality, which primarily is due to late presentation of symptoms and typically advanced disease stage at the time of diagnosis (2). Therefore, we need to improve our methods for diagnosing, detecting, and treating pancreatic cancer.

## Current and Emerging Treatment Trends for PC

The current treatment paradigm for PC involves surgery, radiotherapy, and chemotherapy (2, 4). Operative resection is still the preferred treatment for resectable tumors. Advancement in surgical and imaging technology likely contributed to a slight decrease in PC mortality in the early 2010's (2). In 1996, the first line treatment for patients with metastatic PC included gemcitabine (5). Combinational studies using gemcitabine with other agents failed to improve survival further until *nab*-paclitaxel was added (6, 7), which increased the median overall survival by 1.7 months compared to gemcitabine alone. However, this combination regimen has toxicity which excludes PC patients that have a poor performance status (6, 7). Another treatment option for PC is FOLFIRINOX (5-fluorouracil, irinotecan, and oxaliplatin), which resulted in a 4.3-month survival benefit compared to gemcitabine alone (8). These two treatment options, FOLFIRNOX and gem/*nab*-p, are the current best therapies until disease progression. Second-line treatment options include nanoliposomal irinotecan and 5-FU (approved in 2015), which improved median overall survival by 1.9 months compared to 5-FU alone (9).

Emerging treatment options for PC patients includes tumor microenvironment targeting (including immunotherapies), gene therapy, and PARP inhibitors. All immunotherapies are still in the clinical trial phase, with the most advanced trial involving CXCessoR4, a combination study with anti-CXCR4 (chemokine receptor) and anti-PD-1 (programmed cell death protein, an immune checkpoint inhibitor) (6, 10). In an open-label phase 1b study in patients that had disease progression while under treatment, combinatory therapy with a CC-chemokine receptor 2 (CCR2) kinase antagonist and FOLFIRINOX produced a tumor response in 49% of patients (6). A gene delivery system to deliver wild type p53 (SGT-53) into tumor cells is currently being tested in combination with gem/*nab*-p (6, 11). PARP inhibitors inactivate the repair mechanism for single-stranded DNA breaks (12, 13). These inhibitors induce cell death in tumors, and are given in combination with DNA-damaging agents. Clinical trials are currently underway for all of these emerging treatments for PC. For many of these novel therapeutic regimens, a highly-predictive preclinical model of PC might be helpful to assess and/or optimize the regimen prior to a clinical trial, which theoretically could reduce the risk of a failed clinical trial, thus decreasing (i) cost of drug development and (ii) strain on clinical resources. That is, a highly-predictive preclinical model of PC could streamline the drug development pipeline.

## Current Animal Modeling of PC

Similar to many human diseases, the study of PC has been aided by the use of genetically-edited murine models. Hallmark genetic mutations that drive the progression of PC have been well characterized (14–19). Oncogenic *KRAS* activation has been observed in 95% of PC patients, with 99% of point mutations occurring at the G12 position (20). Murine models have been utilized to study *KRAS* and other genes involved with PC progression, including *TP53, SMAD4*, and *CDKN2A* (14, 18, 19, 21). Expression of the mutant *KRAS*^G12D^ in mice produced metastatic pancreatic tumors; duration of survival in these subjects decreased further with *TP53* antagonism (22). *TP53* is a well-known tumor suppressor that promotes apoptosis in response to cellular stress and DNA damage, and is mutated in 70% of PC patients (20). Furthermore, deletion of tumor suppressor genes (*SMAD4* or *CDKN2A*) enhanced tumor growth in a *KRAS*^G12D^ murine pancreatic cancer model (23, 24).

Despite the progress in genetically-edited murine PC models, a basic issue persists in regard to the mouse's relative ability to recapitulate human disease, including progression of PC and response to therapy. The magnitude of this issue is difficult to quantity using the current biomedical literature, in which many laboratories are heavily invested in the utilization of murine models. To be clear, it is not the intent of this article to criticize or discourage the use of mice in biomedical research, but rather to echo other voices which have questioned the predictive ability of murine models (25–27), and to propose alternative solutions. There has been some indirect evidence of murine fallibility in modeling human disease in the low regulatory approval rate for therapeutics that actually have reached the clinical trial stage, which has been in the range of 5–8% (28, 29). There are many factors that contribute to this low drug approval rate, but one likely reason is the less-than-optimal predictive ability of some murine models (e.g., tumor xenografting into immunosuppressed mice) to determine the efficacy of various therapeutics in humans (30–37).

Rodents may not accurately reflect human biology due to differences in physiology, anatomy, immune response, and genetic sequence (26, 30, 31, 36). For example, there are a number of genes for which the genotype-phenotype correlation is different between mice and humans (Table 1). One of these genes is *APC*^+/−^, in which the human phenotype includes colorectal polyposis (leading to colorectal cancer); the murine *APC*^+/−^ mutant, however, develops small intestinal polyps. In addition, current genetically-edited murine models of cancer have limited tumor heterogeneity and low intratumor mutation rates (43–45), which could limit the clinical relevance of these models and their ability to study tumor immunity and immunotherapy (45, 46). And finally, there is a practical limitation to using murine models in preclinical research: size. Specifically, the development of clinically-relevant diagnostic or interventional technology often is not feasible with murine models due to their small size.

**Table 1:** Comparison of phenotypes from the same genetic mutations between mice, pigs, and humans.

**Mutated gene**	**Murine phenotype**	**Porcine phenotype**	**Human phenotype**
*APC* (38)	Small intestine polyps	Colorectal polyps	Colorectal polyps
*CFTR* (39, 40)	Intestinal disease	Cystic fibrosis	Cystic fibrosis
*TP53* (41)	Axial skeleton tumors	Long bone tumors	Long bone tumors
*DMD* (42)	No phenotype	Progressive muscular dystrophy	Progressive muscular dystrophy

In fairness, murine models are being continually refined for cancer research, including genetically-engineered mouse models (GEMMs) as described above, mice with humanized immune systems (i.e., immunodeficient mice engrafted with human hematopoietic stem cells), and *in vivo* site-directed CRISPR/Cas9 gene-edited mice (25, 31, 47–49). Bacterial microbiota models also have been utilized to demonstrate the effects of bacteria on cancer development and progression in murine models; however the role of the microbiome has not yet been studied in large animal models of cancer (50). Though promising, these more sophisticated murine models come with increased cost and complexity, and experience with them is still early. There remains a need for improved animal models of PC, including potential alternatives to mice, to better predict the human response to anti-cancer therapy. In addition, possession of an animal model of PC with human-sized organs would be helpful in regards to developing specific diagnostic and/or interventional technologies.

## Rationale for a Large Animal Model of PC

As implied above, the rationale for utilizing a large animal model to study PC is to (i) have a platform for research and development of diagnostic/ therapeutic technologies that would not be feasible in murine models, and (ii) to have a highly-predictive preclinical model in which emerging anti-cancer therapies could be vetted and optimized prior to clinical trial. Some current large animal models that are used for biomedical research include non-human primates, dogs, and pigs. Non-human primates are the most “human-like,” but there are societal and ethical concerns involved with the use of these animals for research (51, 52). Similarly, utilization of dogs in biomedical research also can bring up social concerns due to their role as companion animals (53). However, secondary to their relatively long life expectancy as companions, dogs have had some utility in the study of treatments for natural/inherent (i.e., age associated) tumors, including mammary carcinoma, prostate carcinoma, lymphoma, and various sarcomas (54).

Due to their size similarity with humans, various strains of pig have been used for years in biomedical research to develop and refine surgical equipment, instrumentation, and techniques (55). In addition, swine have greater similarity to humans with respect to genomic, epigenetic, physiological, metabolic, and immunological characteristics when compared to the mouse-human similarities (56–60). Generally speaking, the homology between the human and porcine genome is greater than the homology between the human and murine genome. A quantitative indicator of this genomic homology is difficult to generate and depends on the chosen endpoints, a discussion of which is beyond the scope of this review (55). However, these homologies have been estimated at 80–90% (human-porcine) and 60–70% (human-murine) (56, 61–63). Porcine models have been utilized to study a wide range of fields, including physiology, trauma, wound healing, and atherosclerosis (55, 59, 64). Along with primates, swine have been a favored model to study transplantation (65). Human-pig concordance with regard to genotype-phenotype correlation is generally better than human-mouse concordance (Table 1). For example, the CFTR^−/−^ and APC^+/−^ mutants have the same basic phenotype in swine as in humans (38–40). Of note, a porcine genome map was generated in 2012, and further coverage, annotation, and confirmation is ongoing (60, 63, 66). Genetic manipulation of pigs (including knockouts, tissue-specific transgenics, inducible expression, and CRISPR editing), formerly done mostly in mice, has become more routine, with new gene-edited porcine models emerging for diseases such as atherosclerosis, cystic fibrosis, Duchenne muscular dystrophy, and ataxia telangiectasia (67–70).

Use of porcine models would offer other specific advantages. An animal research as large and robust as a pig would permit the testing of multiple, concurrent, clinically-relevant interventions, such as surgery, catheter-directed therapy, systemic chemotherapy, and/or radiotherapy; such combinatory interventions would have questionable feasibility in mice. Regarding the potential to study tumor biomarkers, the relatively large blood volume of a porcine PC model would allow for multiple blood samples to be drawn from the same pig during tumor development (a luxury not possible with the mouse), so precise timing and quantification of biomarker appearance could be correlated with tumor stage. This capability is not possible with a rodent model. On a similar note, immunotherapy study in a porcine PC model would be facilitated by the ability to obtain sufficient quantities of tumor-exposed immune cells that could be conditioned for re-infusion, e.g., as an autologous tumor-specific immunotherapy (71, 72). Furthermore, a porcine PC model could provide clinically-relevant tumor size/burden that would enable development and refinement of technologies to image and localize tumor for diagnosis, treatment, and surveillance (73). The relative size of the porcine subjects also would facilitate the sharing of tissue and blood sample with other investigators to a greater degree that could be accomplished with rodents. This effect would increase the potential number of investigators that could participate, the number of research protocols that could benefit, and the total amount of data that could be produced per research subject.

Of course, there are some caveats in using pigs to study PC. Specifically, the disadvantages of using a porcine model of PC with respect to a murine model include: (i) *Husbandry and Cost*. Depending on the swine strain utilized, the research subject could become quite large (>100 kg) if a prolonged (>1 year) latency is required for tumor development. Specialized equipment and experience would be necessary to handle such subjects. Husbandry is generally more cumbersome and expensive with swine as compared to mice. (ii) *Biosafety*. Biosafety issues, particularly when working with recombinant DNA technology, become more complex when the subject is a pig that is house in a pen, as opposed to a mouse inside a microisolator. (iii) *Aged Subject Availability*. While it is possible to work with aged murine subjects, and even elderly canine companion subjects, this is not really practical with swine, which potentially have a 20–30 year lifespan. Housing pigs for decades would be impractical, costly, and difficult, primarily due to the relatively large size of the mature subject (>150 kg for many strains). (iv) *Reagents and Tools*. Although use of swine in biomedical research has been growing, the availability of reagents and molecular tools specific for swine is not at the same level of availability that exists for mice. For example, the general availability of antibodies specific for porcine antigens is less than that for murine and human antigens. While difficult to quantify, in general this deficiency in porcine research is slowly improving. Of note, some anti-human antibodies will cross-react with porcine antibodies, but this has to be determined on a case-by-case basis. Secondary to these and/or other issues, it may not be practical or desirable for some research laboratories to utilize porcine models.

## A Transgenic Approach to Porcine PC Modeling: the Oncopig Cancer Model

In 2012, the University of Illinois and the NSRRC (National Swine Resource and Research Center, nsrrc.missouri.edu) engineered a Cre-inducible swine model (the “Oncopig;” mini-pig background) (74) which carries an LSL-cassette containing dominant negative *TP53* (R167H mutation) and activated *KRAS* (G12D mutation); i.e., the porcine analog of the KRAS/p53 mouse (22). This Cre-inducible system allows for the expression of both mutations in any cell within the pig. Upon addition of adenovirus expressing Cre recombinase (AdCre) to cultured Oncopig fibroblasts, expression of both mutant *KRAS* and *TP53* was noted (74). The transformed fibroblasts had a shorter cell cycle length and demonstrated *in vitro* “tumorigenic” properties (increased cell migration, soft agar colony formation) and formation of tumors when injected into immunocompromised mice (74). Injection of AdCre into the subcutaneous/intramuscular regions of the Oncopig resulted in tumor formation with pleomorphic features (74). This transgenic pig hence became known as the Oncopig Cancer Model (OCM).

Primary pancreatic ductal cells were cultured from the OCM and then infected with AdCre; these epithelial cells also displayed a transformed phenotype *in vitro*, and expressed mutant *KRAS* and *TP53* (75). These transformed epithelial cells were injected into SCID mice and formed subcutaneous tumors that were histologically and phenotypically similar to human pancreatic ductal adenocarcinoma (PDAC) (75). *In vivo* injection of AdCre directly into the main pancreatic duct of an Oncopig resulted in several nodular tumors after 12 months. Comparison of tumor induced in the OCM pancreas with human PDAC revealed similar morphological features, including a dense desmoplastic stromal reaction that is one key hallmark features of human PDAC (75). In addition, increased expression of proliferative markers (ERK and PCNA) was present in the OCM pancreatic tumor (75).

Key features of modeling PC with the OCM include: (1) the initial tumor induction is genetically defined; (2) the induced tumor is autochthonous; (3) the host has an intact immune system, which is capable of producing an anti-tumor immune response similar to humans, for studying immunotherapies (76); and (4) the tumor induction procedure (AdCre injection) is relatively simple and safe. However, there are some potential issues, such as specificity. Injection of AdCre theoretically could result in non-specific infection of multiple cell types, producing a pleomorphic tumor which could detract from the clinical relevance of the model. There also may an issue of tumor latency with pancreatic tumor in the OCM; in the initial report (75), pancreatic tumor formation required 12 months, and this was not visible on computed tomography nor was it clinically apparent. So, further refinement of the OCM for PC studies might be beneficial.

## Orthotopic Approach: Transformed Porcine PDECs

In contrast to the autochthonous mechanism of tumor induction that the OCM provides, an orthotopic method of tumor induction involves seeding of tumorigenic cells into the pancreas, preferably into an immunocompetent host. In pursuit of this model type, primary cultures of porcine pancreatic ductal epithelial cells (PDECs) were established from explants of normal pancreatic tissue; IHC for cytokeratin-19 in early-passage strains were consistent with epithelial origin of the cultured cells (77). Strains of PDECs subsequently were infected with a lentiviral vector containing GFP, *TP53*^R167H^, and *KRAS*^G12D^ (LV-GKP; generated using porcine sequences), producing clones with demonstrable expression of mutant p53 and KRAS; refer to Table 2 (77). Initial *in vitro* tumorigenic assays of these clones (denoted as PGKP, for PDECs transformed with LV-GKP) demonstrated increases in migration and soft agar colony formation relative to primary PDECs (77). To further increase the transformed phenotype of the PGKP cells, RNAi of SMAD4 and CDKN2A were added using additional LV vectors, with ~70–90% knockdown (77). Relative to primary cells, these secondary clones (PKGPS and PGKPSC) also displayed increased proliferation, soft agar colony formation, invasion, and migration, i.e., evidence of *in vitro* “tumorigenicity” (77), with perhaps enhanced capabilities compared to the primary clone (PGKP cells). The three types of transformed PDECs (summarized in Table 2) were then implanted subcutaneously in nude mice; all three cell lines formed tumors and demonstrated equivalent *in vivo* tumorigenicity (77). In summary, PDEC-derived tumorigenic cell lines were established, which currently are undergoing orthotopic implantation into syngeneic, immunocompetent domestic swine.

**Table 2:** Characteristics of transformed porcine ductal epithelial cells [data published as preprint (77)].

	**KRAS^**G12D**^**	**p53^**R167H**^**	**SMAD4 shRNA**	**p16^**Ink4A**^ shRNA**	**Colony formation**	**Proliferation**	**Migration**	**Invasion**	**Xenografts**
PGKP	+	+			+++				+
PGKPS	+	+	+		++	+++	+	++	+
PGKPSC	+	+	+	+	+++	+++	+	++	+

*P, porcine epithelial cells; G, GFP; K, KRAS^G12D^; P, p53^R167H^; S, SMAD4; C, CDKN2A/p16. Transformed phenotypes of porcine pancreatic ductal epithelial cells in vitro and in vivo. Scale of transformation +++ > ++ > +*.

In terms of generating pancreatic tumor, the theoretical advantages of transformed PDEC implantation over AdCre injection in the OCM include: (i) *Specificity*. the former technique only involves transformed pancreatic ductal cells, meaning that tumor induced with transformed PDEC implantation would be more likely to originate from a specific cell type than tumor induced with AdCre injection in the OCM. (ii) *Target Flexibility*. Cell implantation permits the investigator to choose the targets by which transformation will be accomplished, instead of being restricted to mutant *KRAS* and *TP53*, as in the OCM. (iii) *Host Flexibility*. The investigator can choose the background strain of pig (or another species altogether) with cell implantation, while the OCM by definition involves one transgenic genotype. (iv) *Cost*. The purchase price of OCM subjects likely will be greater compared to most strains of research-quality pigs (though this cost differential becomes less of an issue in the face of multiple months of housing that these experiments would require).

On the other hand, the potential disadvantages of transformed PDEC implantation with respect to AdCre injection in the OCM include: (i) *Immune Rejection*. If allogeneic transformed PDECs are implanted, then there is the possibility that the host would reject the transplanted material (this issue might be minimized by utilizing syngeneic or autologous PDECs). (ii) *Simplicity*. AdCre injection into the OCM is straightforward and has potentially fewer Biosafety issues, as compared to pancreatic harvest, primary cell culture, and numerous viral transformations required for the PDEC implantation technique. (iii) *Local Environment*. As discussed above, tumor induction in the OCM is autochthonous, and likely does not involve local traumatic disruption of tissue architecture which presumably ensues when a cellular suspension is injected. However, the amount and biological relevance of local architecture disruption in these models is not known at this time.

## Applications and Impact

The availability of a validated, genetically-defined porcine model of PC would have multiple potential applications, including (in no particular order):

1. Development and refinement of catheter-based technologies for diagnosis and/or intervention.
2. Discovery and study of serum tumor biomarkers (“liquid biopsy” technology).
3. A preclinical trial tool: a penultimate platform to test novel chemotherapeutic agents that were screened in murine models, prior to pushing a nascent therapy into an expensive clinical trial.
4. A platform for the testing of multiple, concurrent, clinically-relevant interventions, such as surgery, catheter-directed therapy, systemic chemotherapy, and/or radiotherapy (as described under the Rationale section).
5. Study of early events in tumor initiation and progression in an animal subject with a relatively high degree of genetic, physiological, metabolic, immune, and anatomic similarity with humans.
6. Detailed study of tumor heterogeneity (facilitated by a relatively large tumor specimen).
7. Study of the interactions and effects of the microbiome on tumor biology.
8. Development and refinement of tumor-visualization aids (such as fluorescent tumor agents) to assist with R0 resection in surgery.
9. Development and refinement of tools for open and minimally invasive surgery.
10. Refinement of existing imaging tools (such as MRI-based technologies) to diagnosis early stage tumors.
11. Development of novel tumor imaging tools.
12. An educational tool to instruct trainees in surgical resection techniques.

The primary impact of such a porcine PC model would be to increase the efficiency and safety at which impactful technologies and therapies could be brought into the clinical realm. For example, the anti-tumor effect and toxicity of a new chemotherapeutic regimen could be vetted in the porcine model, which could promote (or eliminate) the regimen's introduction into a clinical trial; this screening step likely would increase the probability of success for the human study. As another example, the feasibility, safety, and utility of a catheter-directed energy source in the treatment of PC could be accomplished in a porcine model without ever having to place a patient at risk. Another impact of a porcine model of PC would be an increased understanding of the molecular and cellular biology of the disease in an animal model that would have more relevance than the mouse.

## Conclusion and Future Directions

Current murine models of PC have been tremendously helpful in the progression of understanding and treatment for this disease, but there is an ongoing issue of the relative predictive ability of these murine models. The issue of modeling accuracy likely has contributed in part to an unacceptably high failure rate of experimental therapeutics in clinical trials. Utilizing pigs to model PC has potential benefits, including relevant subject size, increased genetic homology, and better immunological/metabolic mimicry with respect to humans. Specifically, the size of pigs allows for improvement upon imaging and surgical techniques which is not possible with rodents. The OCM has already demonstrated that pancreatic tumor can be induced in the pig with histopathological features similar to human PC. This PDAC model will provide ways for improving early detection, imaging, and surgical techniques of PDAC by following the disease after a defined induction point. Even though the current OCM does have some limitations due to the amount of time it takes to develop tumors, this model potentially could be refined to accelerate tumor growth; for example, by introducing additional edits within the Cre-recombinated cells that would inhibit DNA repair and promote genomic instability, or by generating a tissue-specific inducible promoter for targeted initiation of cellular transformation upon AdCre administration. Another approach to generate a porcine PC model has been orthotopic implantation of transformed PDECs into the pancreas of the syngeneic, immunocompetent pigs. Additional approaches to pancreatic tumor induction in the pig might include direct pancreatic infection with viral vectors containing key tumor-associated gene sequences, *in vivo* CRISPR editing, or combinations of two or more of the technologies described herein. To address the issue of tumor induction in relatively young subjects, diet-induced metabolic syndrome could be used as an adjunctive measure, which likely would increase the physiological age of the subject (and mimic a common clinical co-morbidity). Work remains to be done in the development and validation of a tractable porcine model of PC. Once established, however, a porcine PC model should be a useful addition to the armamentarium of the PC researcher, and should be able to augment and/or complement work done with established murine models.

## Author Contributions

All authors listed have made a substantial, direct and intellectual contribution to the work, and approved it for publication.

### Conflict of Interest Statement

The authors declare that the research was conducted in the absence of any commercial or financial relationships that could be construed as a potential conflict of interest.
